# Activity and Safety of Tegafur, Gimeracil, and Oteracil Potassium for Nasopharyngeal Carcinoma: A Systematic Review and Meta-Analysis

**DOI:** 10.1155/2021/6690275

**Published:** 2021-03-23

**Authors:** Ximing Zhang, Xiumei Tian, Yuezi Wei, Hao Deng, Lichun Ma, Ziyang Chen

**Affiliations:** ^1^School of Basic Medical Sciences, Guangzhou Medical University, Guangzhou 511436, China; ^2^Department of Biomedical Engineering, School of Basic Medical Sciences, Guangzhou Medical University, Guangzhou 511436, China; ^3^Affiliated Stomatology Hospital of Guangzhou Medical University, Guangzhou 511436, China; ^4^Department of Preventive Medicine, School of Public Health, Guangzhou Medical University, Guangzhou 511436, China

## Abstract

In clinical practice, tegafur, gimeracil, and oteracil potassium (S-1) therapy is commonly administered to treat nasopharyngeal carcinoma (NPC). However, its efficacy and safety remain controversial in both randomized controlled trials (RCTs) and non-RCTs. We aimed to evaluate the efficacy and safety of S-1 treatment for NPC. We searched PubMed, Ovid, EMBASE, the Cochrane Library, China National Knowledge Infrastructure, Wanfang Database, and VIP databases for RCTs of chemotherapy with or without S-1 for NPC, from 2001 to 2020. A meta-analysis was performed using RevMan5.3 and Stata15. Randomized controlled trials published in journals were included irrespective of blinding and language used. Patients were diagnosed with NPC through a clinicopathological examination; patients of all cancer stages and ages were included. Overall, 25 trials and 1858 patients were included. There were significant differences in the complete remission (OR = 2.42, 95% CI (1.88–3.10), *P* < 0.05) and overall response rate (OR = 2.68, 95% CI (2.08–3.45), *P* < 0.05) between the S-1 and non-S-1 groups. However, there was no significant difference in partial remission (OR = 1.10, 95% CI (0.87–1.39), *P*=0.42) and seven adverse reactions (leukopenia, thrombocytopenia, nausea and vomiting, diarrhea, dermatitis, oral mucositis, and anemia) between the S-1 and non-S-1 groups. Additionally, statistical analyses with six subgroups were performed. S-1 was found to be a satisfactory chemotherapeutic agent combined with radiotherapy, intravenous chemotherapy, or chemoradiotherapy for NPC. As an oral medicine, the adverse reactions of S-1, especially gastrointestinal reactions, can be tolerated by patients, thereby optimizing their quality of life. S-1 may be a better choice for the treatment of NPC. This trial is registered with CRD42019122041.

## 1. Introduction

Nasopharyngeal carcinoma (NPC) is a malignant tumor prevalent in Southeast Asia and South China. Most patients with NPC are in an advanced stage at diagnosis, as it is asymptomatic in the early stages, resulting in a high mortality rate [1, 2]. Although advanced radiotherapy [3] and concurrent chemotherapy can improve progression-free survival and overall survival [4] in patients with NPC, there is a high risk of local recurrence, distant metastasis, and mortality [5]. Thus, to optimize their quality of life, the efficacy of chemotherapy must be evaluated. However, studies on the efficacy of chemotherapy in randomized controlled trials (RCTs) for NPC have received limited attention [6, 7].

The combination of tegafur, gimeracil, and oteracil potassium (S-1) was approved as a treatment for progressive or recurrent head and neck malignancies in 2001, and it is widely used in the treatment of gastric, esophageal, colorectal, pancreatic, and nonsmall cell lung cancers, and other malignant tumors [8–13]. As a second-generation fluorouracil and oral chemotherapy compound, S-1 has been widely used in clinical applications. Owing to its short half-life and few adverse reactions, S-1 is easy to administer and reduces the pain of intravenous fluids, thereby making patients more receptive, more tolerant, and less likely to develop drug resistance [14–18]. S-1 combined with chemotherapy or radiotherapy for head and neck tumors could have a good effect [19–21]. Moreover, when used during radiotherapy, S-1 has been associated with a low recurrence rate, satisfactory long treatment effects, and improved survival quality [9, 20]. In contrast, its efficacy with chemotherapy is not satisfactory [22] and remains controversial. Blanchard [4] confirmed that chemotherapy combined with radiotherapy significantly improves the survival of patients with locally advanced NPC. Recently, despite the efficacy of S-1 for NPC, as reported by several studies [2, 18, 23], there have been only a few systemic reviews and meta-analyses because of some research limitations. First, the number of patients treated with S-1 is small because the studies are mostly from endemic areas. Second, systematic reviews of RCTs determining the efficacy of S-1 for NPC are limited. The effects of S-1 combined with radiotherapy have been discussed in a previous meta-analysis [24], but those of chemoradiotherapy or chemotherapy alone have not been investigated. Additionally, the databases used [24] only contain data up to 2015; thus, they may have missed data for the past 7 years. It is crucial to systematically analyze the efficacy to improve available chemotherapy approaches that use S-1. Therefore, to objectively evaluate the efficacy and safety of S-1 in the treatment of NPC, we conducted a systematic review and meta-analysis of RCTs of S-1 treatment combined with radiotherapy, intravenous chemotherapy, or chemoradiotherapy in patients with NPC.

## 2. Materials and Methods

### 2.1. Literature Selection

We searched PubMed, Ovid, EMBASE, the Cochrane Library, China National Knowledge Infrastructure, Wanfang Database, and VIP databases. The search language was not restricted, and the retrieval time spanned from January 1, 2001 (S-1 was approved for the treatment of progressive or recurrent head and neck malignancies in 2001) to February 24, 2020. We used the following keywords: S-1, tegafur, gimeracil, oteracil potassium, nasopharyngeal cancer, nasopharyngeal carcinoma, nasopharyngeal cancers, and the Chinese terms for S-1 and NPC.

### 2.2. Eligibility Criteria

RCTs published in journals were included irrespective of blinding. Patients were diagnosed with NPC through a clinicopathological examination, and patients of all cancer stages and ages were included. The experimental group patients were treated with S-1, whereas the control group patients were treated with non-S-1 (e.g., 5-fluorouracil (5-Fu) + cisplatin (DDP), intensity-modulated radiotherapy (IMRT), chemotherapy, or docetaxel (TXT) + DDP) (Table S1 in Supplementary Materials). The baseline characteristics were matched and were comparable between the experimental and control groups in 25 RCTs.

The following studies were excluded: (1) non-RCTs, (2) nonpublished studies, (3) studies with incomplete or unavailable data, and (4) retrospective trials, animal studies, meeting abstracts, letters, comments, editorials, reviews, those not described as RCTs, or systematic reviews and meta-analysis. The retrieved literature was independently screened by two authors (Deng and Ma) using the inclusion and exclusion criteria.

### 2.3. Outcome Measures

The primary outcomes were complete remission (CR), partial remission (PR), and overall response rate (RR) of the short-term effects, whereas the secondary outcomes were adverse reactions. Additionally, statistical analyses with six subgroups were performed. The RR was calculated as follows:

(1) $$\begin{array}{c} \text{RR}=\frac{\text{PR}+\text{CR}}{\text{overall cases}}\times 100. \end{array}$$

### 2.4. Data Collection and Quality Assessment

The data extracted included the first authors' name, time of publication, patient status, trial design, intervention, sample size, dose, outcome indicator, and last follow-up (missing). The extracted information was then cross-verified. Any indeterminate difference or disagreement was resolved through a discussion with the third author who made the final decision.

The quality of the studies was evaluated independently by two authors using the bias risk assessment tool from the RCT evaluation manual in the Cochrane Handbook (version 5.1.0., 2011) [25] with the following parameters: (1) random allocation methods, (2) allocation concealment, (3) blinding of research subjects and researchers, (4) blinding of outcome assessment, (5) incomplete outcome data, (6) selective report outcomes, and (7) other sources of bias. Each included study was evaluated according to the criteria as “high risk,” “low risk,” or “unclear.” When there were disputes regarding the evaluations, a third author made the final decision.

### 2.5. Data Analysis

RevMan (version 5.3, Cochrane Collaboration, Copenhagen, Denmark) and STATA (version 15.0, StataCorp LLC, College Station, TX, USA) software were used for the meta-analysis. If the results were statistically homogeneous (*P* > 0.05, *I*^2^ <50%), a fixed-effect model was selected. If the data were statistically heterogeneous, a random effect model was used. Descriptive analysis was conducted for the data that could not be combined. The Mantel–Haenszel test was used to calculate 95% confidence interval (CIs), and odds ratio (OR) was used to combine the effect. We used the corresponding data model to combine effect sizes across studies and implement a sensitivity analysis for assessing the potential effects of individual datasets on the results and pooled data. Publication bias was identified and was visually inspected using Begg's rank correlation method and the shear complement test.

### 2.6. Protocol and Registration

The protocol was fully implemented in accordance with the requirements of the Preferred Reporting Items for Systematic Reviews and Meta-Analyses [26] (Table S2. PRISMA 2009 checklist in the Supplementary Materials). The review protocol is available on the PROSPERO official website (registration number: CRD42019122041).

## 3. Results

### 3.1. Literature Selection and Study Characteristics

**Figure 1:**
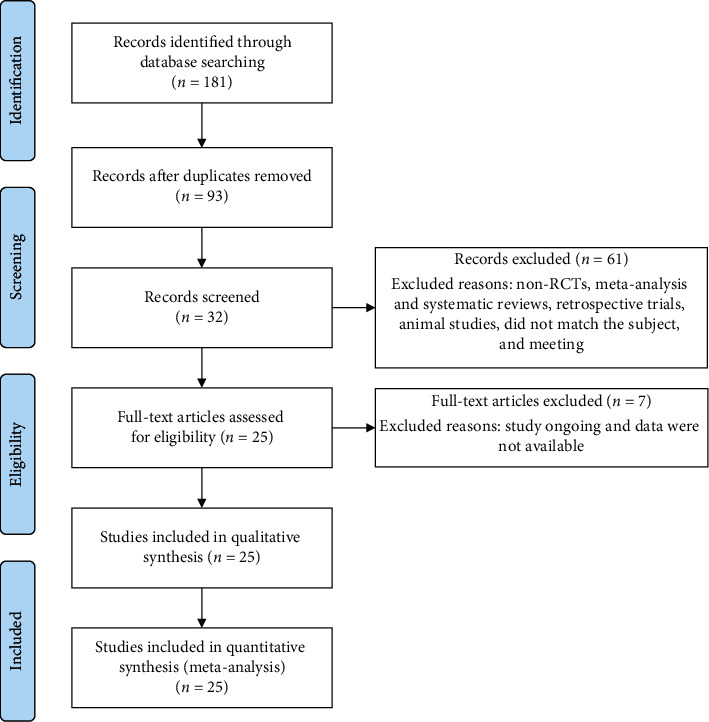
PRISMA flow diagram of literature screening.

One hundred and eighty-one articles were initially retrieved, and the selection process is shown in Figure 1. After removing the duplicates, 93 studies remained. After screening the titles and abstracts, 32 studies remained. After examining the full text of 32 articles, 25 studies [1, 9, 14–16, 19, 22, 27–44] were included in this meta-analysis, and seven were excluded. Twenty-three studies reported three main outcomes (CR, PR, and RR). Patients included in the studies were over 14 years of age; the total number of patients in each study ranged from 40 to 120. Furthermore, 13 studies described patients with NPC in locally advanced stages, 8 in advanced stages, and 3 in early stages. The characteristics of the included studies are summarized in Table S1 in the Supplementary Materials. The 25 RCTs [1, 9, 14–16, 19, 22, 27–44] included involved 1858 patients. Of the studies, only one study used a single-blind method, whereas the others did not describe their allocation concealment or the blinding method. Data from 22 patients were incomplete or lost to follow-up. The outcome data were complete, and most studies did not selectively report results. Two studies had a random sequence generation bias, and allocation concealment in 25 studies was unclear. Five studies had incomplete main outcome data, and one study had a selective reporting bias, although the bias risk was not high. The bias risk assessment is shown in Figure S1 in the Supplementary Materials.

### 3.2. Outcome Measures

#### 3.2.1. Primary Outcome Measures

**Figure 2:**
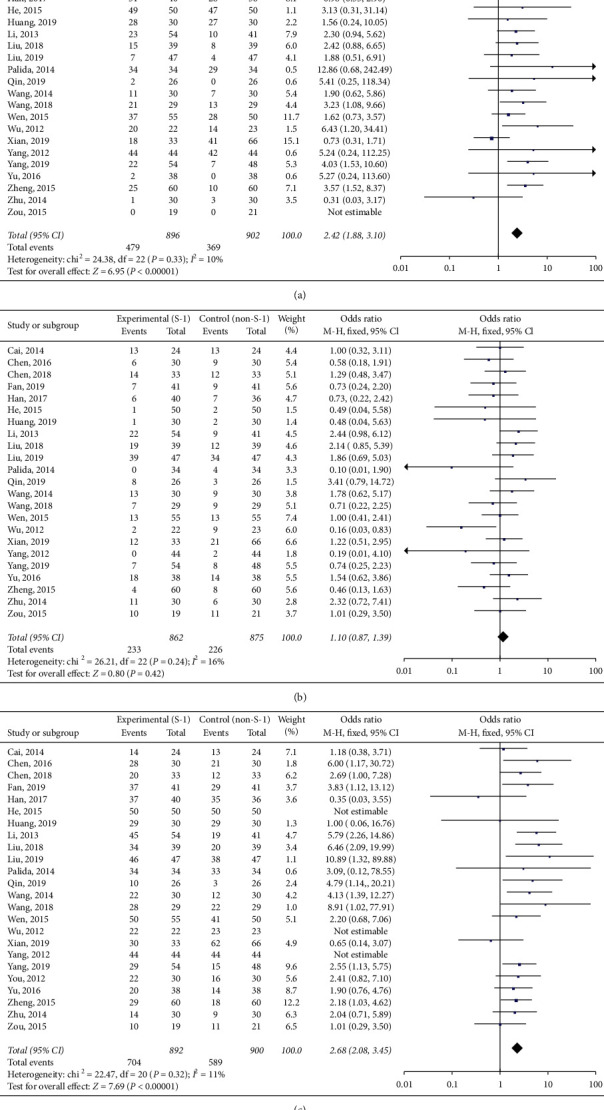
Forest plots of the comparison between the experimental (S-1 treatment) and control (non-S-1 treatment) groups in terms of (a) complete remission (CR), (b) partial remission (PR), and (c) response rate (RR).

CR was reported by 24 studies involving 1798 patients [1, 9, 14–16, 22, 27–44]. The heterogeneity test indicated that the fixed-effect model could be selected (*P*=0.33, *I*^2^ = 10%). The pooled analysis indicated a significant difference between the treatment and control groups (OR = 2.42, 95% CI (1.88–3.10), *P* < 0.05) (Figure 2(a)).

Twenty-three studies involving 1737 patients [1, 9, 14–16, 22, 27, 28, 30–44] reported PR. Based on the heterogeneity test results (*P*=0.24, *I*^2^ = 16%), a fixed-effect model was selected. There was no significant difference between the treatment and control groups (OR = 1.10, 95% CI (0.87–1.39), *P* > 0.05) (Figure 2(b)).

RR was reported by 24 studies involving 1792 patients [1, 9, 14–16, 19, 22, 27, 28, 30–44]. The fixed-effect model was used because there was no statistical heterogeneity (*P*=0.32, *I*^2^ = 11%). There was a significant difference between the treatment and control groups (OR = 2.68, 95% CI (2.08–3.45), *P* < 0.05) (Figure 2(c)).

#### 3.2.2. Secondary Outcome Measures

**Figure 3:**
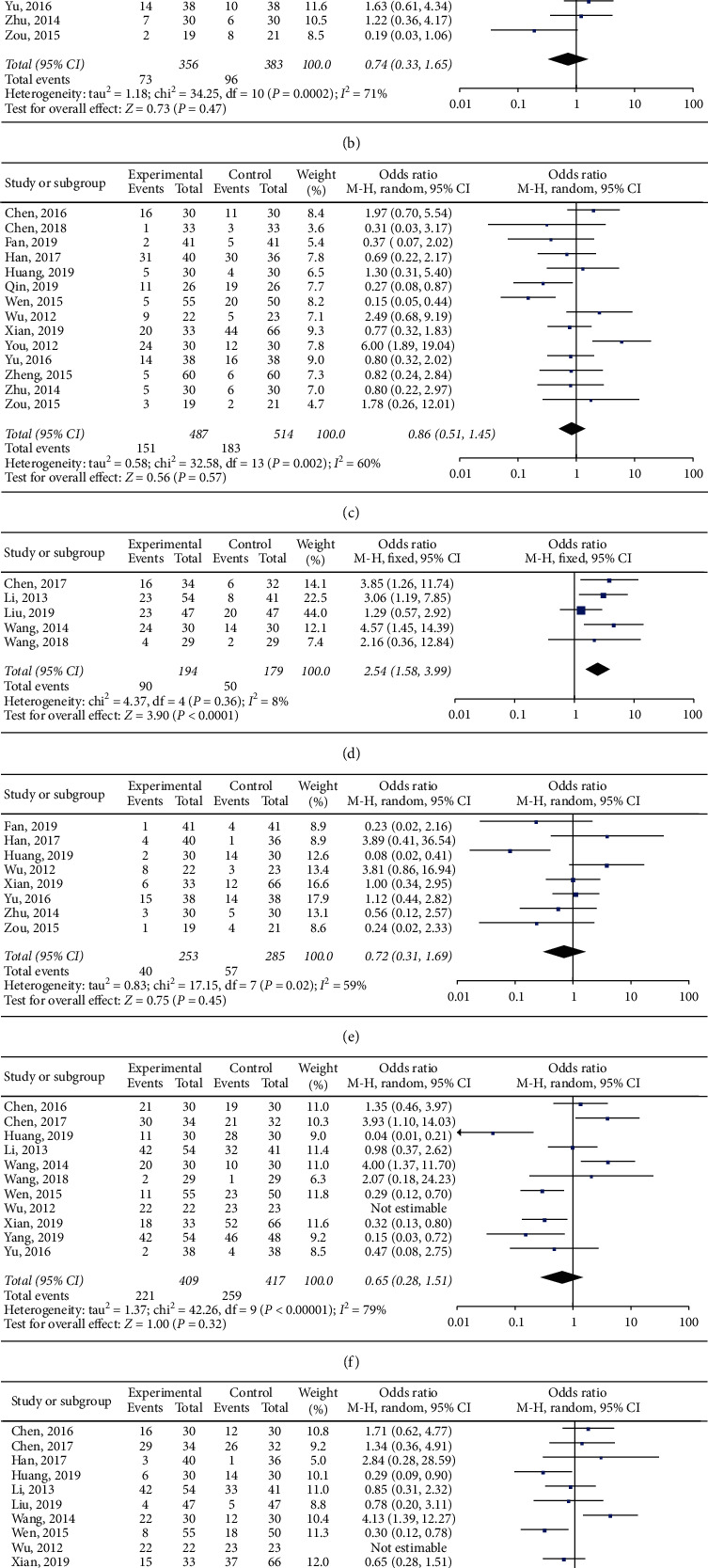
Forest plots of the comparison of adverse reactions between the experimental (S-1 treatment) and control (non-S-1 treatment) groups. (a) Leukopenia, (b) thrombocytopenia, (c) nausea and vomiting, (d) gastrointestinal reactions, (e) diarrhea, (f) oral mucositis, (g) dermatitis, and (h) anemia.

Twelve studies involving 838 patients reported leukopenia [1, 9, 14–16, 19, 30, 32, 38, 41, 42, 44]. The forest plot (Figure 3(a)) revealed no significant difference between the groups (OR = 1.03, *P* > 0.05). As there was statistical heterogeneity (*P* < 0.05, *I*^2^ = 70%), the random-effect model was selected.

Thrombocytopenia was reported by 11 studies involving 739 patients [1, 9, 14–16, 22, 30, 32, 41, 43, 44]. The random effect model was selected based on the heterogeneity test results (*P* < 0.05, *I*^2^ = 71%). There was no significant difference between the groups (OR = 0.74, *P* > 0.05) (Figure 3(b)).

Fourteen studies [1, 9, 14–16, 19, 22, 30, 32, 36, 41–44] involving 1001 patients reported nausea and vomiting. The random-effect model was selected based on the heterogeneity test results (*P* < 0.05, *I*^2^ = 60%). The forest plot (Figure 3(c)) revealed that there was no significant difference between the groups (OR = 0.86, *P* > 0.05).

Five studies involving 373 patients reported gastrointestinal reactions [27, 29, 34, 37, 38]. The fixed-effect model was selected based on the heterogeneity test results (*P* > 0.05, *I*^2^ = 8%). There was a significant difference between the groups (OR = 2.51, *P* < 0.05) (Figure 3(d)).

Eight studies [1, 14, 16, 22, 32, 36, 41, 43] involving 538 patients reported diarrhea. The random-effect model was selected based on the heterogeneity test results (*P* < 0.05, *I*^2^ = 59%). There was no significant difference between the treatment and control groups (OR = 0.72, *P* > 0.05) (Figure 3(e)).

Ten studies involving 721 patients reported oral mucositis [14, 15, 22, 27, 29, 32, 37, 38, 40, 41]. The random-effect model was selected based on the heterogeneity test results (*P* < 0.05, *I*^2^ = 79%). There was no significant difference between the treatment and control groups (OR = 0.72, *P* > 0.05) (Figure 3(f)).

Eleven studies involving 862 patients [1, 9, 14, 15, 22, 27, 29, 32, 34, 37, 40] reported dermatitis. The random-effect model was selected based on the heterogeneity test results (*P* < 0.05, *I*^2^ = 68%). There was no significant difference between the groups (OR = 0.77, *P* > 0.05) (Figure 3(g)).

Anemia was reported by 12 studies [1, 9, 14–16, 22, 32, 38, 41–44] involving 851 patients. The random effect model was selected based on the heterogeneity test results (*P* < 0.05, *I*^2^ = 66%). The forest plot (Figure 3(h)) revealed no significant difference between the groups (OR = 0.78, *P* > 0.05).

#### 3.2.3. Subgroup Outcome Measures

Statistical analyses with six subgroups were performed, and the outcome measure was RR. These subgroups were as follows: (1) locally advanced NPC (LANPC), (2) advanced NPC (ANPC), (3) treatment of the experimental and control groups with chemotherapy, (4) treatment with chemotherapy concomitant with radiotherapy (S-1 treatment) and radiotherapy alone (non-S-1 treatment) (RC vs. R), (5) treatment of the experimental and control groups with chemoradiotherapy (RC), and (6) treatment of the experimental group with S-1 and the control group with 5-Fu (S-1 vs. 5-Fu). The sites of locally advanced NPC were different from advanced NPC. Locally advanced NPC is not associated with distant metastasis to the lung and liver, whereas advanced NPC can have distant metastasis to these sites.

**Figure 4:**
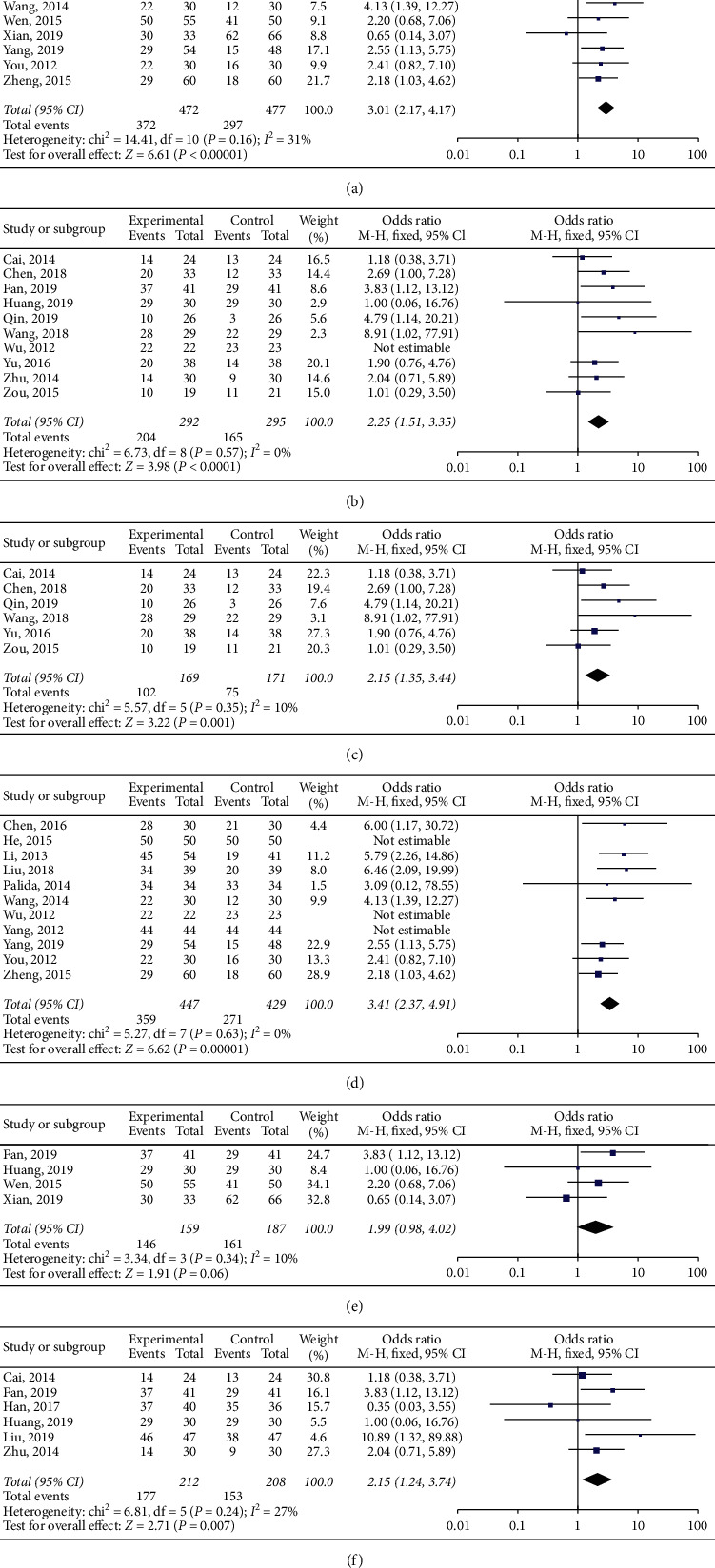
Forest plots of the comparison between S-1 treatment and non-S-1 treatment in the subgroups: (a) locally advanced NPC RR, (b) advanced NPC RR, (c) chemotherapy RR, (d) RC vs R RR, (e) RC RR, and (f) S-1 vs. 5-Fu RR. RR, response rate; chemotherapy, treatment of the experimental and control groups with chemotherapy; RC vs. R, chemotherapy concomitant with radiotherapy (S-1 treatment) and radiotherapy alone (non-S-1 treatment); RC, treatment of the experimental and control groups with chemoradiotherapy; 5-Fu, 5-fluorouracil; S-1 vs. 5-Fu, treatment of the experimental group with S-1 and the control group with 5-Fu.

Thirteen studies involving 1016 patients reported RR to LANPC [1, 9, 14, 15, 19, 22, 27, 32–34, 36, 37, 40]. The fixed-effect model was selected based on the heterogeneity test results (*P*=0.21, *I*^2^ = 23%). There was a significant difference between the treatment and control groups (OR = 3.22, 95% CI (2.28–4.56), *P* < 0.05) (Figure 4(a)).

Eight studies involving 520 patients reported RR to ANPC [16, 28, 30, 38, 41–44]. The fixed-effect model was used because the data were not statistically heterogeneous (*P*=0.59, *I*^2^ = 0%). There was a significant difference between the treatment and control groups (OR = 2.15, 95% CI (1.48–3.11), *P* < 0.05) (Figure 4(b)).

The RR to chemotherapy was reported by six studies involving 340 patients [28, 30, 38, 41, 43, 44]. The fixed-effect model was used because the data were not statistically heterogeneous (*P*=0.35, *I*^2^ = 10%). There was a significant difference between the treatment and control groups (OR = 2.15, 95% CI (1.35–3.44), *P* < 0.05) (Figure 4(c)).

Eleven studies involving 876 patients reported RR to RC vs. R [14, 15, 19, 22, 27, 31, 33, 35, 37, 40, 42]. The fixed-effect model was used because the data were not statistically heterogeneous (*P*=0.63, *I*^2^ = 0%). There was a significant difference between the treatment and control groups (OR = 3.28, 95% CI (2.29–4.69), *P* < 0.05) (Figure 4(d)).

The experimental and control groups' RR to chemoradiotherapy was reported by four studies with 346 patients [9, 22, 32, 36]. The fixed-effect model was used because there was no statistical heterogeneity (*P*=0.34, *I*^2^ = 10%), and there was no significant difference between the treatment and control groups (OR = 1.99, 95% CI (0.98, 4.02), *P*=0.06 > 0.05) (Figure 4(e)).

Six studies involving 420 patients reported RR to S-1 vs. 5-Fu [1, 16, 28, 32, 34, 36]. The fixed-effect model was selected based on the heterogeneity test results (*P*=0.24, *I*^2^ = 27%). There was a significant difference between the treatment and control groups (OR = 2.15, 95% CI (1.24–3.74), *P* < 0.05) (Figure 4(f)).

### 3.3. Sensitivity Analysis

**Figure 5:**
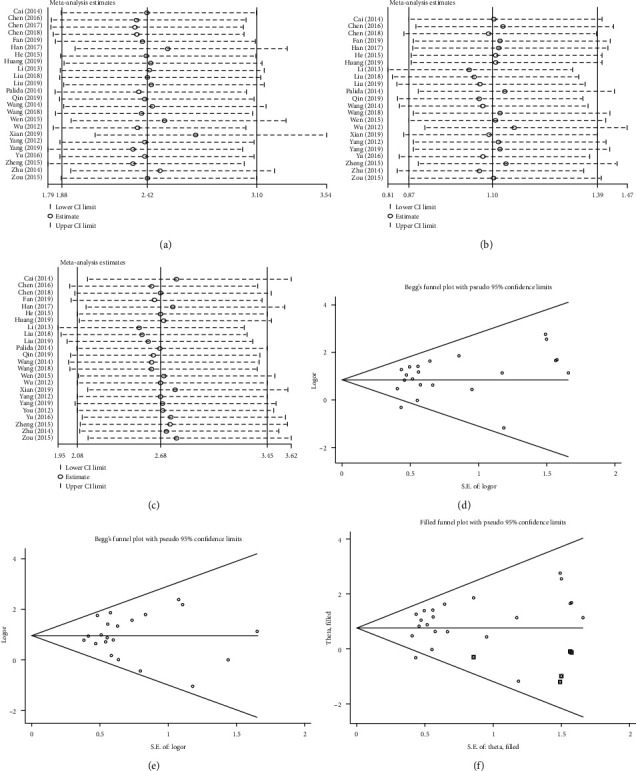
Sensitivity analysis of the primary outcomes: (a) complete remission, (b) partial remission, and (c) response rate. Funnel plot analysis: Begg's test for (d) complete remission and (e) response rate. (f) Trim-and-fill method for complete remission.

By removing one study at a time, the sensitivity analysis was performed to assess the potential effect of individual datasets on the results and pooled data. For CR (Figure 5(a), Table 1, and Table S3 in Supplementary Materials), the OR value in the pooled analysis became stable (*I*^2^ = 0%) after removing the study of Xian [22]. This indicated that the results of the other 24 studies were relatively consistent and that the efficacy of S-1 was certain. For PR and RR (Figures 5(b) and 5(c)), the results were consistent with those of the forest plot; thus, these studies can be considered homogeneous.

### 3.4. Publication Bias

Using Begg's funnel plot and the trim-and-fill method, publication bias was analyzed (Figures 5(d)–5(f)). There was evidence of publication bias in the meta-analysis of CR. After adding the six missing studies (two of which overlapped) according to the trim-and-fill plot (Figure 5(f), Table 2), the results of the meta-analysis did not change significantly. The publication bias was small, indicating that the conclusions were robust. There was no publication bias in the analysis of RR because Begg's funnel plots and the results of the trim-and-fill method were similar (Figure 5(e)) [45].

## 4. Discussion

In this study, 25 RCTs were reviewed to evaluate the efficacy of S-1 treatment for NPC. The control (non-S-1) groups were treated with radiotherapy alone, intravenous chemotherapy, or chemoradiotherapy. Of the 25 studies, 24 indicated that S-1 is more effective than other non-S-1 treatments for NPC. As an oral drug, S-1 is also easier to administer than intravenous chemotherapy regimens and could reduce the incidence of adverse reactions. Xian [22] included three chemotherapeutic control groups (radiotherapy + S-1, radiotherapy + TXT, and radiotherapy + DDP) and concluded that treatment with S-1 is not as effective for NPC; this contradicted the findings of the other 24 RCTs.

We then analyzed the clinical efficacy of S-1 in NPC. There were significant differences in CR and RR but not in PR, indicating that the three primary outcomes are not statistically heterogeneous. Therefore, S-1 is active against NPC.

Just five studies included 1-year [27, 29, 37, 40, 43] and 2-year [9, 19, 27, 37, 40] survival rates. The results of these studies showed that S-1 effectively improved the survival rates of patients with NPC. However, the survival and recurrence rates were low and inconsistent among these studies. For example, Yang [40] reported 1-year and 2-year survival rates, but the recurrence and metastasis rates were not indicated. Yu [41] found that the survival rate, distant transfer rate, and recurrence rate in the S-1 group were significantly higher than those in the control group, whereas the distant metastasis rate and recurrence rate in the study were opposite. The survival rate, distant metastasis rate, and recurrence rate were not specified in several studies. Therefore, some results could not be merged, and the data quality was not optimal. More rigorous studies with a higher number of patients should be conducted to analyze the survival rates.

To analyze the nine adverse reactions of S-1 treatment, we used forest plots. Only the adverse reactions reported in at least five studies were analyzed. Data on the adverse reactions contained four levels (I–IV). There were no significant differences in the occurrence rate of leukopenia, thrombocytopenia, nausea and vomiting, diarrhea, oral mucositis, dermatitis, and anemia between the S-1 and non-S-1 groups, indicating that these adverse reactions are tolerable. However, gastrointestinal reactions were more severe in the S-1 group than in the control group in five RCTs. Nausea, vomiting, and diarrhea are considered gastrointestinal reactions, and some studies reported these adverse reactions. However, these studies only included gastrointestinal reactions without separate detailed items (nausea and vomiting in 14 studies, diarrhea in 8 studies, and gastrointestinal reactions in 5 studies), and therefore, the analyses of nausea and vomiting and diarrhea were probably different from those of gastrointestinal reactions. Importantly, there was heterogeneity in all adverse reactions except for gastrointestinal reactions, which may be related to the stage of NPC, patient age, or the classification of adverse reactions.

Furthermore, we performed statistical analyses with six subgroups. The forest plots revealed significant differences in five subgroups, namely, LANPC, ANPC, chemotherapy, RC vs. R, and S-1 vs. 5-Fu, but not in the subgroup RC. This indicated that the outcomes of the six subgroups are not statistically heterogeneous. Among the 25 RCTs, 22 RCTs had discussed the treatment of S-1 for locally advanced NPC and advanced NPC, and 17 articles had analyzed the treatment of experimental and control groups with chemotherapy and chemotherapy concomitant with radiotherapy (S-1 arms) and radiotherapy alone (non-S-1 arms). The use of S-1 was reported effective in the studies involving these subgroups.

For the chemotherapy subgroup, six studies [28, 30, 38, 41, 43, 44] were included, and all of them were on advanced NPC. This subgroup analysis showed the combined treatment with S-1 for advanced NPC: improved the clinical efficacy [30, 41, 44], toxicities and adverse effects were tolerable [28, 38, 41, 43], safe [30, 41], prolonged survival of patients with NPC, and improved their quality of life [38, 41, 44].

Additionally, for treatment with chemoradiotherapy [9, 22, 32, 36], there were only four studies with the S-1 and control groups. Wen et al. [9] concluded that the efficacy of S-1 and control was similar but superior in terms of toxicity over the control.

Critically, as a kind of 5-Fu derivative oral anticancer drug, S-1 is comprised of tegafur, gimeracil, and oteracil potassium [1, 15, 30, 40]. Tegafur can be transformed into 5-Fu in the human body to exert antitumor activity [1, 30, 38], and the catabolism of 5-Fu is inhibited by gimeracil [15, 29]; thus, a relatively constant blood drug concentration has to be maintained [27, 36]. Oteracil potassium can reduce gastrointestinal adverse reactions [1, 28, 29].

Especially for 5-Fu, among the 25 RCTs, six studies [1, 16, 28, 32, 34, 36] analyzed the effects of S-1 for NPC, four studies were for LANPC [1, 32, 34, 36], two studies were for ANPC [16, 28], two evaluated chemoradiotherapy [32, 36], two evaluated neoadjuvant chemotherapy [1, 34], and two evaluated chemotherapy [16, 28]. Han et al. [1, 28] reported that S-1 and 5-Fu have equal efficacies. Liu et al. [16, 32, 34, 36] indicated that S-1 was more effective. Cai et al. [28] reported S-1 was better for patients with deep vein catheterization. Furthermore, Zhu et al. reported that there was no phlebitis in the S-1 group [16]. Studies in this subgroup reported the following: toxic and adverse effects were tolerated by patients [28, 32], adverse reactions did not increase [16, 34], and most adverse reactions were at the 0–2 levels [1, 32]. In particular, the studies pointed out that the 1–4 level toxicities of S-1 were lower than those of 5-Fu [32], neoadjuvant chemotherapy of S-1 had effectively improved the immune function of patients with locally advanced NPC [34], and S-1 had reduced the total proportion of adverse reactions [36]. Therefore, S-1 was better than 5-Fu for NPC, and S-1 was safe for treatment [32, 34].

Based on the outcome measures and the above discussion, it can be concluded that treatment with S-1 was more active than that without S-1 for NPC. S-1 was a satisfactory chemotherapeutic agent combined with radiotherapy or intravenous chemotherapy for NPC, importantly. As an oral medicine, the adverse reactions, especially gastrointestinal reactions, associated with S-1 can be tolerated by patients, thereby optimizing the quality of life of patients. S-1 may be a better choice of treatment for NPC.

From the sensitivity analysis, the OR value of CR was stable after removing the study of Xian et al. [22], and the *I*^2^ changed from 10% to 0%. The results indicated that the other 23 studies were relatively consistent. For RR, the included studies were homogeneous. This confirmed the efficacy of S-1. Xian [22] evaluated the efficacy and toxic adverse effects of chemotherapy concurrent with radiotherapy in the treatment of locally advanced NPC in three groups administered TXT, DDP, or S-1. The S-1 treatment presented a slightly lower efficacy than the others, but the incidence of oral mucositis was significantly lower. Except in the study of Xian [22], the efficacy of S-1 for NPC in 23 studies was consistent with the finding of our meta-analysis.

There are some weaknesses to our meta-analysis. We found publication bias in CR; thus, six studies had to be added to the meta-analysis. Additionally, our study used published trials rather than individual patient data, which could lower the accuracy of the estimates. Hence, the results obtained from the literature review and meta-analysis suggest the need to confirm the noninferiority in efficacy of S-1 in different settings with prospective randomized, controlled clinical trials, in which toxicity and quality of life are also evaluated, to better define the role of this drug in NPC treatment. In addition, there is the potential limitation of late toxicities.

## 5. Conclusions

In summary, our study showed that S-1 for NPC treatment has satisfactory activity; moreover, the adverse effects were tolerated by patients. As S-1 is an oral medication, it is convenient for clinical use. S-1 may be a standard regimen for NPC; especially, it is suitable for patients who cannot tolerate the intravenous chemotherapy. Furthermore, it can reduce the pain caused by intravenous administration and thereby improve the quality of life of patients. To confirm the effectiveness of S-1 treatment, more rigorous studies with a higher number of patients should be conducted, and later toxicities in patients, such as the reaction in the gastrointestinal tract after long-term use of S-1, should be analyzed, especially to verify the efficacy in advanced NPC patients with recurrence and metastasis. Additionally, the outcome indicators of these efficacy and toxicities should be further unified.

## Figures and Tables

**Table 1:** Sensitivity analysis of complete remission.

Criterion	Included studies (*n*)	Experimental groups (*n*)	Control groups (*n*)	OR^a^ (95% CI)	*P* value	*I* ^*2*^ (%)
Before exclusion	24	896	902	2.42 (1.88–3.10)	0.33	10
After exclusion	23	863	836	2.72 (2.09–3.54)	0.75	0

^a^Odds ratio.

**Table 2:** Trim-and-fill method for complete remission.

Step 1^a^						

Model	Pooled estimate	95% CI		Asymptotic		
		Lower limit	Upper limit	*z* value	*P* value	Studies (*n*)
Fixed	0.853	0.595	1.111	6.483	0	23
Random	0.866	0.587	1.144	6.096	0	

Test for heterogeneity: *Q* = 24.331 on 22 degrees of freedom (*P*=0.330)						
Moment-based estimate of between-studies variance = 0.043						

Step 2^b^						
Trimming estimator: linear						
Meta-analysis type: random effect model						

Iteration	Estimate	Tn		# to trim		Diff
1	0.866	184		4		276
2	0.796	197		5		26
3	0.79	200		6		6
4	0.784	200		6		0

Step 3^c^						
Filled meta-analysis						

Model	Pooled	95% CI		Asymptotic		Studies (*n*)
	Estimate	Lower limit	Upper limit	*z* value	*P* value	
Fixed	0.78	0.529	1.03	6.104	0	29
Random	0.783	0.515	1.051	5.726	0	

^a^Meta-analysis was performed by combined results of all effects with a fixed model and random model. ^b^The trimming estimator of the random-effect model (through four iterations). ^c^Meta-analysis was performed again after the inclusion of the missing studies.

## Data Availability

The data used to support the findings of this study are included within the article and supplementary information files.

## Supplementary Materials

Supplementary Materials Table S1. Basic characteristics of the included studies. Table S2. PRISMA checklist. Table S3. Sensitivity analysis for complete remission. Table S4. Search strategies. Figure S1. Results of the risk bias assessment of 25 RCTs (EPS 4462 kb).
