# Supplementary material for: Activity and Safety of Tegafur, Gimeracil, and Oteracil Potassium for Nasopharyngeal Carcinoma: A Systematic Review and Meta-Analysis
Source: J Oncol. 2021 Mar 23;2021:6690275. doi: 10.1155/2021/6690275 (PMC8009729; doi:10.1155/2021/6690275)
Supplement: Supplementary Materials — Table S1. Basic characteristics of the included studies. Table S2. PRISMA checklist. Table S3. Sensitivity analysis for complete remission. Table S4. Search strategies. Figure S1. Results of the risk bias assessment of 25 RCTs (EPS 4462 kb). [file 6690275.f1.docx]

**Supplementary Materials**

Table S1: Basic characteristics of the included studies

| Study | n (T/C) | Age (T/C) | Stage | Treatment | Control | Outcome | | L/D |
| --- | --- | --- | --- | --- | --- | --- | --- | --- |
|  |  |  |  |  |  | MO | ADR |  |
| Cai [28] | 24/24 | 25–74/ 27–72 | Advanced | DDP+S-1 (40–60 mg, bid × 14 d with 7-d interval, 21 d/1 cycle, and 2 cycles in total) | 5-Fu+DDP | 1,2,3 |  | 0 |
| Chen [15] | 30/30 | 25–65 | Locally advanced | IGRT+lobaplatin+S-1 (80 mg/d·m^2^, twice daily, bid × 14 d with 7-d interval, 21 d/1 cycle, and 3 cycles in total) | IGRT | 1,2,3 | 1,2,3,6,7,8 | 0 |
| Chen [29] | 34/32 | 73.27±2.12/ 72.96±2.45 | Locally advanced | IMRT+S-1 (60 mg/m^2^, twice daily, bid × 28 d with 14-d interval, 42 d/1 cycle, continue to take 1 cycle after IMRT) | IMRT | 1 | 4,6,7 | 0 |
| Chen [30] | 33/33 | 37–71/35–73 | Advanced | Chemotherapy, TXT+S-1 (40 mg/d·m^2^, twice daily, bid × 28 d with 14-d interval, 42 d/1 cycle) | Chemotherapy, TXT+DDP | 1,2,3 | 1,2,3 | 0 |
| Fan [36] | 41/41 | 2–69/19–66 | Locally advanced | DDP+IMRT+S-1 (60 mg/m^2^·d, 21 d/1 cycle, and 2 cycles in total) | 5-Fu+DDP+IMRT | 1,2,3 | 3,5 | 0 |
| Han [1] | T:40/36, C:37/35 | (16–65) 47±4.4/ 44.9±9.1 | Locally Advanced | Neoadjuvant chemotherapy (NC), TXT+DDP+S-1 (60 mg/d·m^2^, twice daily, bid × 14 d), DDP+IMRT | NC, TXT+DDP+5-Fu, DDP+IMRT | 1,2,3 | 1,2,3,5,7,8 | 4 |
| He [31] | 50/50 | 24–66/22–65 | Early stage | Radiotherapy+S-1 (120 mg/d·m^2^, twice daily, bid × 14 d with 7-d interval, 21 d/1 cycle, and 2 cycles in total) | Radiotherapy | 1,2,3 |  | 0 |
| Huang [32] | 30/30 | 18–65 | Locally advanced | DDP+IMRT+S-1 (60 mg/d·m^2^, twice daily, bid × 14 d) | DDP+IMRT+5-Fu | 1,2,3 | 1,2,3,5,6,7,8 | 0 |
| Li [27] | 54/41 | 68–84/67–82 | Locally advanced | Radiotherapy+S-1 (120 mg/d·m^2^, twice daily, bid × 28 d with 14-d interval, 42 d/1 cycle, and 4 cycles in total) | Radiotherapy | 1,2,3 | 4,6,7 | 5 |
| Liu [33] | 39/39 | 62-78/64–75 | Locally advanced | IMRT+S-1 (100 mg/d·m^2^, twice daily, bid × 28 d) | IMRT | 1,2,3 |  | 0 |
| Liu [34] | 47/47 | 34–74/25–77 | Locally advanced | Induction chemotherapy (ICT), TXT+DDP+S-1 (60 mg/d·m^2^, twice daily, bid × 14 d with 7-d interval, 21 d/1 cycle, and 6 cycles in total). | ICT, TXT+DDP+5-Fu | 1,2,3 | 4,7 | 0 |
| Palida [35] | 34/34 | 16–78/15–81 | Early stage | 3DCRT+S-1 (120 mg/d·m^2^, twice daily, bid × 14 d with 7-d interval until the end of radiotherapy) | 3DCRT | 1,2,3 |  | 0 |
| Qin [44] | 26/26 | 37–78/39–80 | Advanced | TXT+S-1 (40 mg/d, bid × 14 d with 7-d interval, 21 d/1 cycle, 2 cycles) | DDP+TXT | 1,2,3 | 1,2,3,8 | 0 |
| Wang [37] | 30/30 | 35–62/36–60 | Locally advanced | IMRT+S-1 (80 mg/d·m^2^, twice daily, bid × 14 d with 7-d interval until the end of radiotherapy) | IMRT | 1,2,3 | 4,6,7 | 0 |
| Wang [38] | 29/29 | 37–68/35–70 | Advanced | Routine chemotherapy (RC)+S-1 (40 mg/d·m^2^, bid × 14 d with 7-d interval, 21 d/1 cycle, and 6 cycles in total), | RC+TXT | 1,2,3 | 1,4,6,8 | 0 |
| Wen [9] | 55/50 | 25–68/20–69 | Locally advanced | CCRT+S‑1 (40–60 mg, twice daily for 4 consecutive weeks) | CCRT+DDP | 1,2,3 | 1,2,3,7,8 | 2 |
| Wu [14] | 22/23 | 32–73 | Locally Advanced | Radiotherapy+S-1 (80 mg/d·m^2^, twice daily, bid × 28 d with 14-d interval, 42 d/1 cycles, continued for 2 cycles after radiotherapy) | Radiotherapy | 1,2,3 | 1,2,3,5,6,7,8 | 0 |
| Xian [22] | 33/66 | 20–65 | Locally advanced | Radiotherapy+S-1 (40 mg/d·m^2^, twice daily, bid × 14 d with 7-d interval, 21 d/1 cycles, a total of 2–3 cycles) | Radiotherapy+TXT/DDP | 1,2,3 | 2,3,5,6,7,8 | 0 |
| Yang [39] | 44/44 | 15–78 | Early stage | 3DCRT+S-1 (60 mg, twice daily, bid × 14 d with 7-d interval until the end of radiotherapy) | 3DCRT | 1,2,3 |  | 6 |
| Yang [40] | 54/48 | 62–81/63–82 | Locally advanced | IMRT+S-1 (60 mg, twice daily, bid × 28 d) | IMRT | 1,2,3 | 6,7 | 0 |
| You [19] | 30/30  (27/28) | Age ≥70 | Locally advanced | Radiotherapy+S-1 (60 mg, twice daily, bid × 28 d with 14-d interval, 42 d/1 cycle, a total of 4 cycles) | Radiotherapy | 3 | 1,3 | 5 |
| Yu [41] | 38/38 | 33–77/34–74 | Advanced | TXT+S-1 (40 mg/d·m^2^, bid × 14 d with 7-d interval, 21 d/1 cycle, 6 cycles) | TXT+DDP | 1,2,3 | 1,2,3,5,6,8 | 0 |
| Zheng [42] | 60/60 | 66–75/65–75 | Advanced | IMRT+S-1 (40–60 mg, 1 month) | IMRT | 1,2,3 | 1,3,8 | 0 |
| Zhu [16] | 30/30 | 60–71 | Advanced | S-1 (40–60 mg, twice daily, bid × 14 d with 7-d interval, 21 d/1 cycle, 2 cycles in total) | 5-Fu | 1,2,3 | 1,2,3,5,8 | 0 |
| Zou [43] | 19/21 | 24–74 | Advanced | NDP+S-1 (80–120 mg/d, twice daily, bid × 14 d with 7-d interval, 21 d/1 cycle, 2 cycles in total) | GEM | 1,2,3 | 2,3,5,8 | 0 |

Outcomes: MO, main outcome; 1 is complete remission (CR), 2 is partial remission (PR), and 3 is overall response rate (RR). ADR is adverse reaction, and 1–9 corresponds to leucopenia, thrombocytopenia, nausea and vomiting, gastrointestinal reaction, diarrhea, oral mucosa, dermatitis, decreased hemoglobin, and anemia. T/C is treatment/control groups; L/D is the number of lost follow-up/deletion cases; TXT is docetaxel; DDP is cisplatin; NDP is nedaplatin; GEM is gemcitabine; Fu is fluorouracil; IGRT is image-guided radiation therapy; IMRT is intensity-modulated radiation therapy; 3DCRT is 3D conformal radiation therapy, CCRT is concurrent chemoradiation therapy.

Table S2: PRISMA checklist

| **Section/topic** | | **#** | **Checklist item** | | **Reported on page #** |
| --- | --- | --- | --- | --- | --- |
| **TITLE** | | | | |  |
| Title | | 1 | Identify the report as a systematic review, meta-analysis, or both. | | 1 |
| **ABSTRACT** | | | | |  |
| Structured summary | | 2 | Provide a structured summary including, as applicable: background; objectives; data sources; study eligibility criteria, participants, and interventions; study appraisal and synthesis methods; results; limitations; conclusions and implications of key findings; systematic review registration number. | | 1 |
| **INTRODUCTION** | | | | |  |
| Rationale | | 3 | Describe the rationale for the review in the context of what is already known. | | 2 |
| Objectives | | 4 | Provide an explicit statement of questions being addressed with reference to participants, interventions, comparisons, outcomes, and study design (PICOS). | | 2 |
| **METHODS** | | | | |  |
| Protocol and registration | | 5 | Indicate if a review protocol exists, if and where it can be accessed (e.g., Web address), and, if available, provide registration information including registration number. | | 1,4 |
| Eligibility criteria | | 6 | Specify study characteristics (e.g., PICOS, length of follow-up) and report characteristics (e.g., years considered, language, publication status) used as criteria for eligibility, giving rationale. | | 3–4 |
| Information sources | | 7 | Describe all information sources (e.g., databases with dates of coverage, contact with study authors to identify additional studies) in the search and date last searched. | | 2–3 |
| Search | | 8 | Present full electronic search strategy for at least one database, including any limits used, such that it could be repeated. | | Table S4 |
| Study selection | | 9 | State the process for selecting studies (i.e., screening, eligibility, included in systematic review, and, if applicable, included in the meta-analysis). | | 2–3 |
| Data collection process | | 10 | Describe method of data extraction from reports (e.g., piloted forms, independently, in duplicate) and any processes for obtaining and confirming data from investigators. | | 3–4 |
| Data items | | 11 | List and define all variables for which data were sought (e.g., PICOS, funding sources) and any assumptions and simplifications made. | | 3–4 |
| Risk of bias in individual studies | | 12 | Describe methods used for assessing risk of bias of individual studies (including specification of whether this was done at the study or outcome level), and how this information is to be used in any data synthesis. | | 2–4, Figure S1 |
| Summary measures | | 13 | State the principal summary measures (e.g., risk ratio, difference in means). | | 3 |
| Synthesis of results | | 14 | Describe the methods of handling data and combining results of studies, if done, including measures of consistency (e.g., I^2^) for each meta-analysis. | | 3 |
| Risk of bias across studies | 15 | | | Specify any assessment of risk of bias that may affect the cumulative evidence (e.g., publication bias, selective reporting within studies). | Not applicable |
| Additional analyses | 16 | | | Describe methods of additional analyses (e.g., sensitivity or subgroup analyses, meta-regression), if done, indicating which were pre-specified. | 6-11 |
| **RESULTS** | | | | |  |
| Study selection | 17 | | | Give numbers of studies screened, assessed for eligibility, and included in the review, with reasons for exclusions at each stage, ideally with a flow diagram. | 4–5, Figure 1 |
| Study characteristics | 18 | | | For each study, present characteristics for which data were extracted (e.g., study size, PICOS, follow-up period) and provide the citations. | 3, Table S1 |
| Risk of bias within studies | 19 | | | Present data on risk of bias of each study and, if available, any outcome level assessment (see item 12). | 11–12, Figure S1 |
| Results of individual studies | 20 | | | For all outcomes considered (benefits or harms), present, for each study: (a) simple summary data for each intervention group (b) effect estimates and confidence intervals, ideally with a forest plot. | 5–10, Figures 2–4 |
| Synthesis of results | 21 | | | Present results of each meta-analysis done, including confidence intervals and measures of consistency. | 5–11, Figures 2–5 |
| Risk of bias across studies | 22 | | | Present results of any assessment of risk of bias across studies (see Item 15). | Not applicable |
| Additional analysis | 23 | | | Give results of additional analyses, if done (e.g., sensitivity or subgroup analyses, meta-regression [see Item 16]). | 8–12, Figure 5, Tables 1–2, Table S3 |
| **DISCUSSION** | | | | |  |
| Summary of evidence | 24 | | | Summarize the main findings including the strength of evidence for each main outcome; consider their relevance to key groups (e.g., healthcare providers, users, and policy makers). | 12–14 |
| Limitations | 25 | | | Discuss limitations at study and outcome level (e.g., risk of bias), and at review-level (e.g., incomplete retrieval of identified research, reporting bias). | 14 |
| Conclusions | 26 | | | Provide a general interpretation of the results in the context of other evidence, and implications for future research. | 15 |
| **FUNDING** | | | | |  |
| Funding | 27 | | | Describe sources of funding for the systematic review and other support (e.g., supply of data); role of funders for the systematic review. | 15 |

*From:* Moher D, Liberati A, Tetzlaff J, Altman DG, The PRISMA Group (2009). Preferred Reporting Items for Systematic Reviews and Meta-Analyses: The PRISMA Statement. PLoS Med 6(6): e1000097. doi:10.1371/journal.pmed1000097

For more information, visit: www.prisma-statement.org.

Table S3: Sensitivity analysis for complete remission

| Study | | Estimate | [95% CI] | |
| --- | --- | --- | --- | --- |
| Cai | [28] | 2.4134183 | 1.8798965 | 3.0983555 |
| Chen | [15] | 2.3483355 | 1.8180431 | 3.0333054 |
| Chen | [29] | 2.3392665 | 1.8136337 | 3.0172398 |
| Chen | [30] | 2.351155 | 1.8293368 | 3.021822 |
| Fan | [36] | 2.3863447 | 1.84251 | 3.0906975 |
| Han | [1] | 2.5432935 | 1.966738 | 3.2888682 |
| He | [31] | 2.409287 | 1.8752578 | 3.0953951 |
| Huang | [32] | 2.4368002 | 1.8951571 | 3.1332469 |
| Li | [27] | 2.4276364 | 1.8730356 | 3.1464529 |
| Liu | [33] | 2.4172237 | 1.869382 | 3.1256158 |
| Liu | [34] | 2.440671 | 1.8935908 | 3.1458089 |
| Palida | [35] | 2.3636646 | 1.8395205 | 3.0371561 |
| Qin | [44] | 2.4009032 | 1.8697628 | 3.0829237 |
| Wang | [37] | 2.4468765 | 1.8951746 | 3.1591833 |
| Wang | [38] | 2.3804014 | 1.8431872 | 3.0741918 |
| Wen | [9] | 2.5236113 | 1.9401398 | 3.2825541 |
| Wu | [14] | 2.3558424 | 1.8304219 | 3.0320842 |
| Xian | [22] | 2.7176847 | 2.0891469 | 3.5353239 |
| Yang | [39] | 2.4012206 | 1.869997 | 3.0833526 |
| Yang | [40] | 2.3266573 | 1.7973391 | 3.0118604 |
| Yu | [41] | 2.4011474 | 1.8699429 | 3.0832539 |
| Zheng | [42] | 2.3293557 | 1.7948514 | 3.0230346 |
| Zhu | [16] | 2.4945633 | 1.9389549 | 3.2093816 |
| Zou | [43] | 2.4175022 | 1.884491 | 3.1012709 |
| Combined | | 2.4175022 | 1.884491 | 3.1012709 |

Table S4: Search strategies

| **PubMed**  Searched on: May 16, 2020  Results: 13 | |
| --- | --- |
| Search | Query |
| #1 | Search: **("2001/01/01"[Date - Create] : "2020/02/24"[Date - Create])** |
| #2 | Search: **(nasophary*[Title/Abstract]) OR (nasophary*[MeSH Terms])** |
| #3 | Search: **(((tegafur gimeracil oteracil[MeSH Terms]) OR (tegafur gimeracil oteracil[Title/Abstract])) OR (S-1[Title/Abstract])) OR (TS-1[Title/Abstract])** |
| #4 | Search: **(((((clinical[Title/Abstract]) OR (random*[Title/Abstract])) OR (prospective[Title/Abstract])) OR (controlled[Title/Abstract])) OR (multicenter[Title/Abstract])) OR (blind*[Title/Abstract])** |
| #5 | Search: **(clinical trial) OR (((randomized controlled trial) OR (RCT)) OR (RCTs))** |
| #6  (#4OR#5) | Search: **((((((clinical[Title/Abstract]) OR (random*[Title/Abstract])) OR (prospective[Title/Abstract])) OR (controlled[Title/Abstract])) OR (multicenter[Title/Abstract])) OR (blind*[Title/Abstract])) OR ((clinical trial) OR (((randomized controlled trial) OR (RCT)) OR (RCTs)))** |
| #7 | Search: **(animals[MeSH Terms]) NOT (humans[MeSH Terms])** |
| #8 | Search: **((((("2001/01/01"[Date - Create] : "2020/02/24"[Date - Create])) AND ((nasophary*[Title/Abstract]) OR (nasophary*[MeSH Terms]))) AND ((((tegafur gimeracil oteracil[MeSH Terms]) OR (tegafur gimeracil oteracil[Title/Abstract])) OR (S-1[Title/Abstract])) OR (TS-1[Title/Abstract]))) AND (((((((clinical[Title/Abstract]) OR (random*[Title/Abstract])) OR (prospective[Title/Abstract])) OR (controlled[Title/Abstract])) OR (multicenter[Title/Abstract])) OR (blind*[Title/Abstract])) OR ((clinical trial) OR (((randomized controlled trial) OR (RCT)) OR (RCTs))))) NOT ((animals[MeSH Terms]) NOT (humans[MeSH Terms]))** |

The search strategy was adapted as appropriate for searching Ovid, EMBASE, the Cochrane Library, China National Knowledge Infrastructure (CNKI), Wanfang Database, and VIP Database. As specific websites have a variety of search capabilities, the searches were adapted to individual sites.


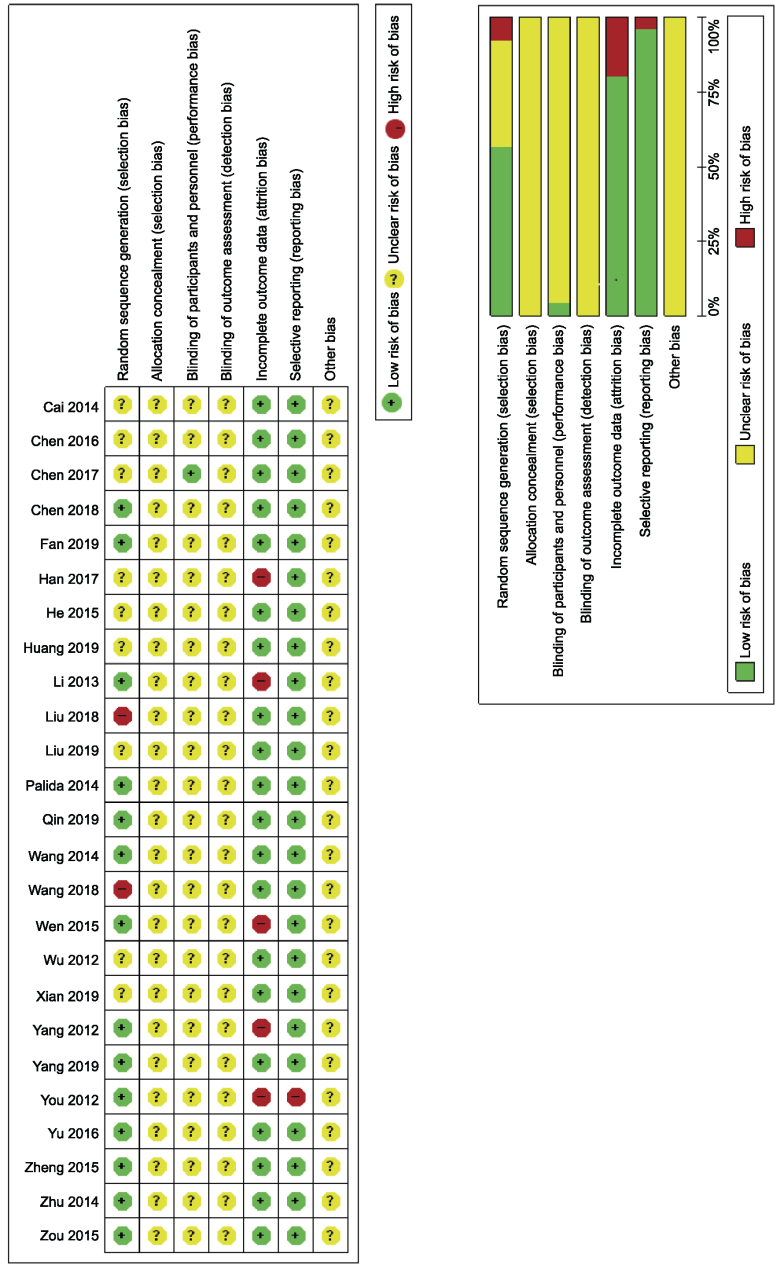


**Figure S1:** Results of risk bias assessment in the 25 randomized controlled trials (EPS 3056 kb)
